# Management of community-acquired brain abscess and intracranial empyema: a survey of UK neurosurgical centres

**DOI:** 10.1007/s10096-026-05522-4

**Published:** 2026-05-04

**Authors:** Carmen Thompson Perea, Holly Roy, James Hatcher, Sophia de Saram, Jacob Bodilsen, Peter Whitfield, William Singleton, Jack Wildman, Michelle M. Kameda-Smith, Eliza Gil

**Affiliations:** 1 Clinical Research Department, London School of Hygiene & Tropical Medicine, London, UK; 2 Department of Neurosurgery, University Hospitals Plymouth NHS Trust, Plymouth, UK; 3 Peninsula Medical School, Faculty of Health, University of Plymouth, Plymouth, UK; 4 Infection, Immunity & Inflammation,Great Ormond Street Institute of Child Health, University College London, London, UK; 5 Department of Microbiology, Virology and Infection Control, Great Ormond Street Hospital for Children, NHS Foundation Trust, London, UK; 6 European Society of Clinical Microbiology and Infectious Diseases Study Group of Infections of the Brain (ESGIB), ESCMID, Basel, Switzerland; 7 Division of Infection, University College London Hospital, London, UK; 8 Department of Infectious Diseases, Aalborg University Hospital, Aalborg, Denmark; 9 Department of Clinical Medicine, Aalborg University, Aalborg, Denmark; 10 Department of Paediatric Neurosurgery, Bristol Royal Hospital for Children, Bristol, UK; 11 Translational Health Sciences, Bristol Medical School, University of Bristol, Bristol, UK; 12 Neurosurgery Department, North Bristol NHS Trust, Bristol, UK; 13 Department of Pediatric Neurosurgery, Oregon Health and Science University, Doernbecher Children’s Hospital, Portland, OR USA; 14 UCL Respiratory, Division of Medicine, University College London, London, UK

**Keywords:** Intracranial infections, Brain abscess, Subdural empyema, Neurosurgical procedures, Anti-bacterial agents

## Abstract

**Purpose:**

We sought to describe current perceptions and attitudes to management of of brain abscess (BA) or sub-/extra-dural empyema (SDE/EDE) in the United Kingdom (UK) to compare this to the 2024 European Society of Clinical Microbiology and Infectious Diseases BA guidelines.

**Methods:**

We conducted a web-based survey of infection specialists (IS) and neurosurgeons (NS) at neurosurgical centres across the UK.

**Results:**

IS from 27/39 (69%) and NS from 18/39 (46%) UK neurosurgical centres participated. All IS reported use of a third-generation cephalosporin as empirical antibiotic therapy, 57/61 (93%) alongside metronidazole, 19/57 (33%) preferring oral metronidazole throughout treatment. Most IS (46/60, 76.7%) consider switching to oral antibiotics prior to completing 6 weeks intravenous (IV) therapy, with 33/46 (71.7%) considering a 1–2 week minimum IV duration if there has been neurosurgical intervention. Most NS (22/25, 88%) agreed that neurosurgical intervention is indicated for any BA *≥* 2.5 cm diameter, most (21/25, 84%) favouring burr hole aspiration. For SDE/EDE only 12/25 (48%) of NS would surgically intervene in all circumstances. Most IS and NS (72/76, 94.7%) would consider intrathecal antibiotics in ruptured BA with ventriculitis; only 11/74 (14%) reported experience with intracavitary antimicrobials. 44/74 (59%) reported using steroids in BA, while 20/74 (27%) reported avoiding steroids. Reimaging was favoured at 2–3 weeks by NS, IS favoured 4–8 weeks, or not reimaging.

**Conclusion:**

There are areas of marked variation in the management of BA and SDE/EDE in the UK, particularly early switch from IV to oral antibiotics, SDE surgery, repeat brain imaging and use of steroids.

**Supplementary Information:**

The online version contains supplementary material available at 10.1007/s10096-026-05522-4.

## Introduction

Intracranial suppurative infections (ICSI) are focal pus collections within the cranium: brain abscess (BA) within the brain parenchyma, subdural empyema (SDE) between the dura and arachnoid mater or extradural empyema (EDE) between the dura mater and skull. These infections are mostly caused by bacteria, with *Streptococcus anginosus* group the dominant bacterial pathogen in community-acquired BA in England [1–3]; less commonly these infections are also caused by mycobacteria, fungi or parasites [4]. These infections require neurosurgical intervention and are associated with significant mortality, estimated around 10%, and morbidity, with 20–40% of patients left with long term neurological impairment [5–8]. While ICSI is uncommon, the incidence appears to be increasing in diverse settings including in England [2, 5, 9–11].

The European Society of Clinical Microbiology and Infectious Diseases (ESCMID) published updated guidelines in 2024 on the diagnosis and management of brain abscess recommending 6–8 weeks of intravenous (IV) antimicrobials (a third-generation cephalosporin alongside metronidazole) for aspirated or conservatively treated BA, with consideration of a shorter duration (e.g. 4 weeks) in excised BA. There is a paucity of randomised controlled trial evidence to support clinical decision making or guideline development in this area. The ESCMID BA guideline duration recommendation was “conditional” and the quality of evidence “low” as there were only 4 sufficiently detailed studies able to be included in the meta-analysis to address the appropriate duration of antimicrobial therapy for bacterial BA [12–15]. There are no national UK guidelines for the antimicrobial or surgical management of ICSI and the published literature, largely composed of case series, suggests diversity in UK clinical practice [16–18].

Given the increasing burden of ICSI and the lack of UK national guideline, we sought to describe current UK perceptions and attitudes to the management of ICSI with a view to supporting future work to develop UK national guidelines and also to allow individual specialists managing these infections to contextualise their practice.

## Methods

A web-based survey (Supplementary Material 1) was designed on the RedCap platform, using similar work as reference where available [19, 20], piloted amongst infection specialists (IS) and neurosurgeons (NS) across three hospital clinical services and refined according to feedback. UK neurosurgical centres were identified from The Society of British Neurological Surgeons (SBNS) and Brain & Spine Foundation lists [21, 22]. All Microbiology departments at UK neurosurgical centres, and Infectious Diseases departments where these could be identified, were contacted by email and invited to nominate *≥* 1 IS to participate in the survey. IS was defined as a medically qualified consultant in medical microbiology and/or infectious diseases. NS were contacted through SBNS. All participants were issued with an individual weblink to access the survey. Up to two reminders were sent to participants in order to maximise completion rates. Blank answers were excluded. Data analysis was performed in Microsoft Excel and GraphPad Prism 10.

**Table 1:** Distribution of survey responses on the management of brain abscesses stratified by specialty

	IS	NS	Total responses
Total	63	25	88
Location			
England	57 (90%)	19 (76%)	76 (86%)
East Midlands	1 (1%)	1 (4%)	2 (2%)
East of England	4 (6%)	2 (8%)	6 (7%)
London	14 (22%)	2 (8%)	16 (21%)
North East England	11 (17%)	0	11 (14%)
North West England	9 (14%)	3 (12%)	12 (15%)
South East	1 (1%)	2 (8%)	3 (3%)
South West	6 (9%)	5 (20%)	11 (14%)
West Midlands	3 (4%)	2 (8%)	5 (6%)
Yorkshire and The Humber	8 (12.7%)	2 (8%)	10 (13%)
Northern Ireland	0	0	0
Republic of Ireland	0	1 (4%)	1 (14%)
Scotland	3 (4%)	4 (16%)	7 (8%)
Wales	3 (4%)	1 (4%)	4 (4%)
Patient cohort			
Adult	22 (34%)	14 (56%)	36 (41%)
Paediatric	17 (27%)	4 (16%)	21 (24%)
Both adults & children	24 (38%)	7 (28%)	31 (35%)
Time in specialty			
< 5 years	4 (6%)	0	4 (5%)
5-10 years	15 (23%)	4 (16%)	19 (22%)
11-20 years	31 (49%)	11 (44%)	42 (48%)
>20 years	13 (20%)	10 (40%)	23 (26%)
Brain abscess cases per year			
1 to 2	8 (12%)	2 (8%)	10 (11%)
3 to 5	5 (7%)	7 (28%)	12 (14%)
More than 5	50 (79%)	16 (64%)	66 (75%)
Subdural empyema cases per year			
1 to 2	6 (9%)	6 (24%)	12 (14%)
3 to 5	12 (19%)	6 (24%)	18 (20%)
More than 5	45 (71%)	13 (52%)	58 (66%)

Data are presented as the number of responses. Percentages represent the proportion of total responses within each specialty and in total. Abbreviations: *IS*, infection specialists; *NS*, neurosurgeons

## Results

### Respondents

**Fig. 1:**
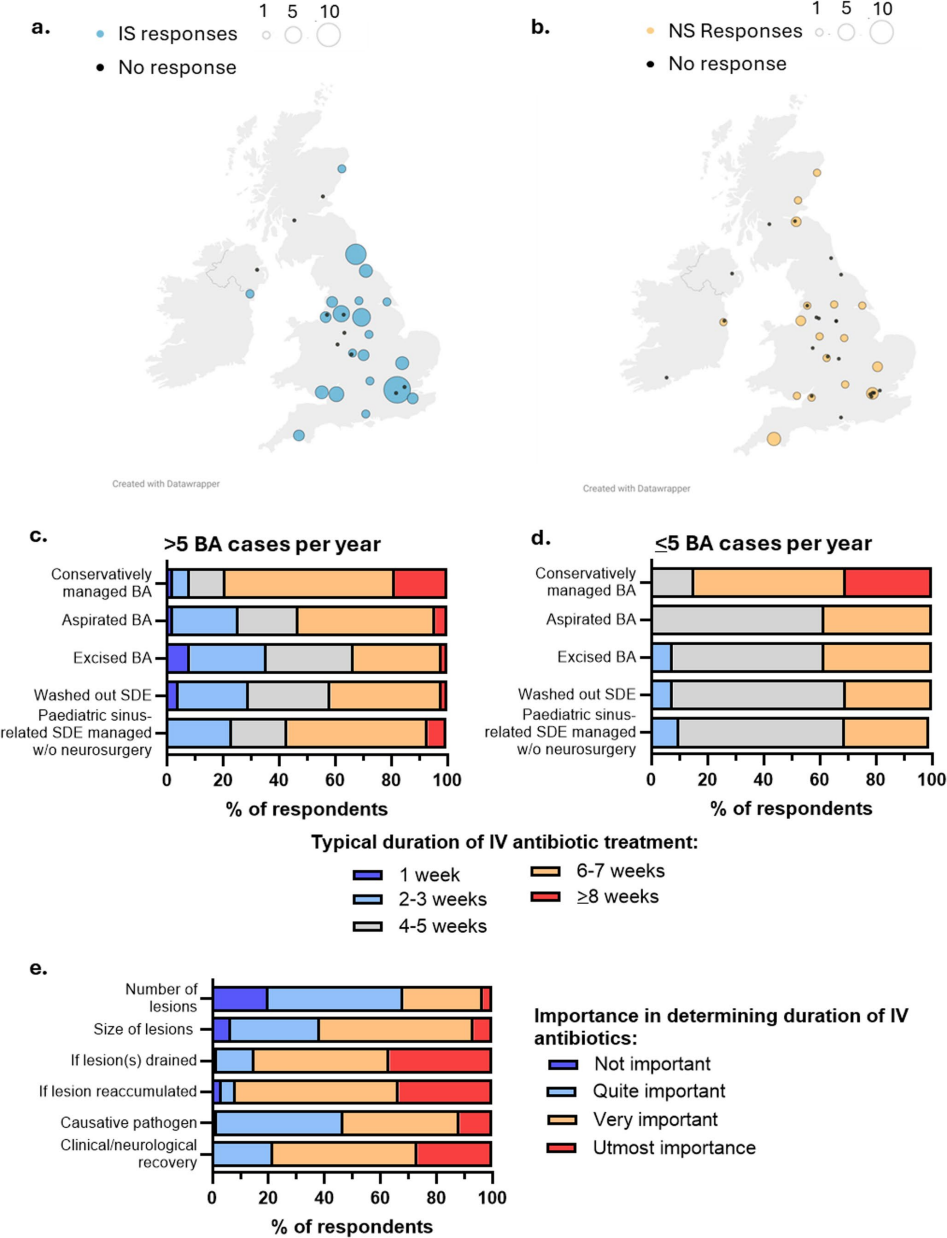
**a** Geographic distribution of survey responses by UK neurosurgical centres amongst infection specialists (IS). **b** Geographic distribution of survey responses by UK neurosurgical centres amongst neurosurgeons (NS). The size of coloured circles is proportional to the number of departmental responses. Black dots indicate centres that were invited to participate but did not respond. Responses from centres within the same city were grouped together. Maps generated using Datawrapper. **c**, **d** Reported duration of antibiotics by clinical brain abscess (BA) or subdural empyema (SDE) scenario for **c** IS reporting seeing > 5 BA cases/year and **d** < 5 BA cases/year. Stacked bars represent the proportion of respondents selecting each duration category for patients with each condition. Percentages correspond to the proportion of total responses **e** Importance of clinical factors in determining intravenous (IV) antibiotic duration for brain abscess. Stacked bars represent the proportion of respondents rating each consideration from “not important” to “utmost importance” when deciding the duration of antibiotic treatment. Percentages correspond to the proportion of total responses

A total of 88 responses were received: 63 IS and 25 NS, with IS responses from 27/39 (69%), and NS responses from 18/39 (46%), of UK neurosurgical centres, as well as a single neurosurgical response from the Republic of Ireland (Fig. 1a, b; Table 1). Of IS, 22/63 (34%) worked exclusively with adults, 17/63 (27%) exclusively with children, and 24/62 (38%) with both; for NS the figures were 14/25 (56%), 4/25 (16%) and 7/25 (28%) respectively. The majority (65/88, 73%) had over ten years specialty experience, with 66/85 (77%) managing more than 5 brain abscess cases annually (Table 1).

### Local approach

16/84 (19%) respondents reported no local antibiotic guidelines and 38/85 (46%) indicated no MDT discussion of cases, although there may be valuable interdisciplinary discussion of cases outside a formal MDT. MDT composition at 23 neurosurgical centres was described. All MDTs (23/23,100%) included a microbiologist, 18/23 (78%) were also attended by an Infectious Disease specialist, 19/23 (82%) were attended by NS, 12/23 (52%) by a radiologist, 5/23 (21.7%) a neurologist and 1/23 (4%) an ENT specialist.

### Choice of empiric antibiotics

All responding IS reported use of a third-generation cephalosporin (9/61 [14%] cefotaxime, 49/61 [80%] ceftriaxone, 3/61 [4%] either cefotaxime or ceftriaxone) as empirical therapy. 57/61 (93%) reported using additional metronidazole, 19/57 (33%) of whom highlighted they would use the oral formulation throughout if the patient could swallow and had no contraindications. 3/61 (4%) reported meropenem as an alternative empiric regimen. 59/60 (98%) reported using outpatient ambulatory intravenous (IV) antibiotic treatment in management of ICSI. 5/61 (8%) reported varying the empirical management of SDE/EDE compared to BA, typically reviewing the need for metronidazole.

### IV antibiotic treatment

In all contexts, some IS reported durations of IV antibiotics as short as 1–3 weeks and others > 8 weeks with duration varying by clinical context. In the context of aspirated BA, 13/61IS (21%) preferred 1–3 weeks of IV antibiotics, 46/61 (75%) preferred 4–7 weeks and 2/61 (3%) preferred *≥* 8 weeks, while in the context of excised BA the values were 18/61(30%), 42/61(69%) and 1/61 (2%) respectively. For a conservatively managed BA, 4/61 IS (7%) preferred 1–3 weeks of IV antibiotics, 44/61 (72%) preferred 4–7 weeks and 13/61(21%) preferred *≥* 8 weeks. IS seeing > 5 BA cases per year more frequently reporting shorter durations of IV antibiotics than those seeing *≤* 5 cases (Fig. 1c, d).

The most cited factor influencing IV antibiotic duration was whether the lesion(s) had been drained (22/60, 36.7%), followed by lesion recurrence (20/60, 33%) and clinical or neurological recovery (17/60, 26.7%) (Fig. 1e). Additional influential factors included MDT discussion, causative pathogen, radiological response and patient factors like aetiology of the infection, immune status, clinical response, oral antibiotic tolerability as well as practical considerations such as IV access and feasibility of outpatient antibiotic therapy (OPAT).

### Oral antibiotic treatment

Most responding IS (46/60, 76.7%) reported switching to oral antibiotics prior to completing 6 weeks IV therapy when a fully sensitive organism was identified, and 28/60 (46.7%) reported doing so even when no organism was identified. Of IS considering an early switch, 33/46 (71.7%) would consider a minimum acceptable IV antibiotic duration to be 1–2 weeks for either an aspirated/excised simple community-acquired BA or a washed-out community acquired SDE in an immunocompetent patient. Most IS (30/46, 65%) who stepped down early determined the oral antibiotic duration based on the preceding IV course, typically aiming for a total treatment duration of 6–8 weeks. A minority of IS (12/59, 20%) reported using oral consolidation therapy after a full IV course, sometimes extending to 2–3 months, while the majority (47/59, 79.7%) did not use any oral antibiotic consolidation.

### Brain abscess surgery

**Fig. 2:**
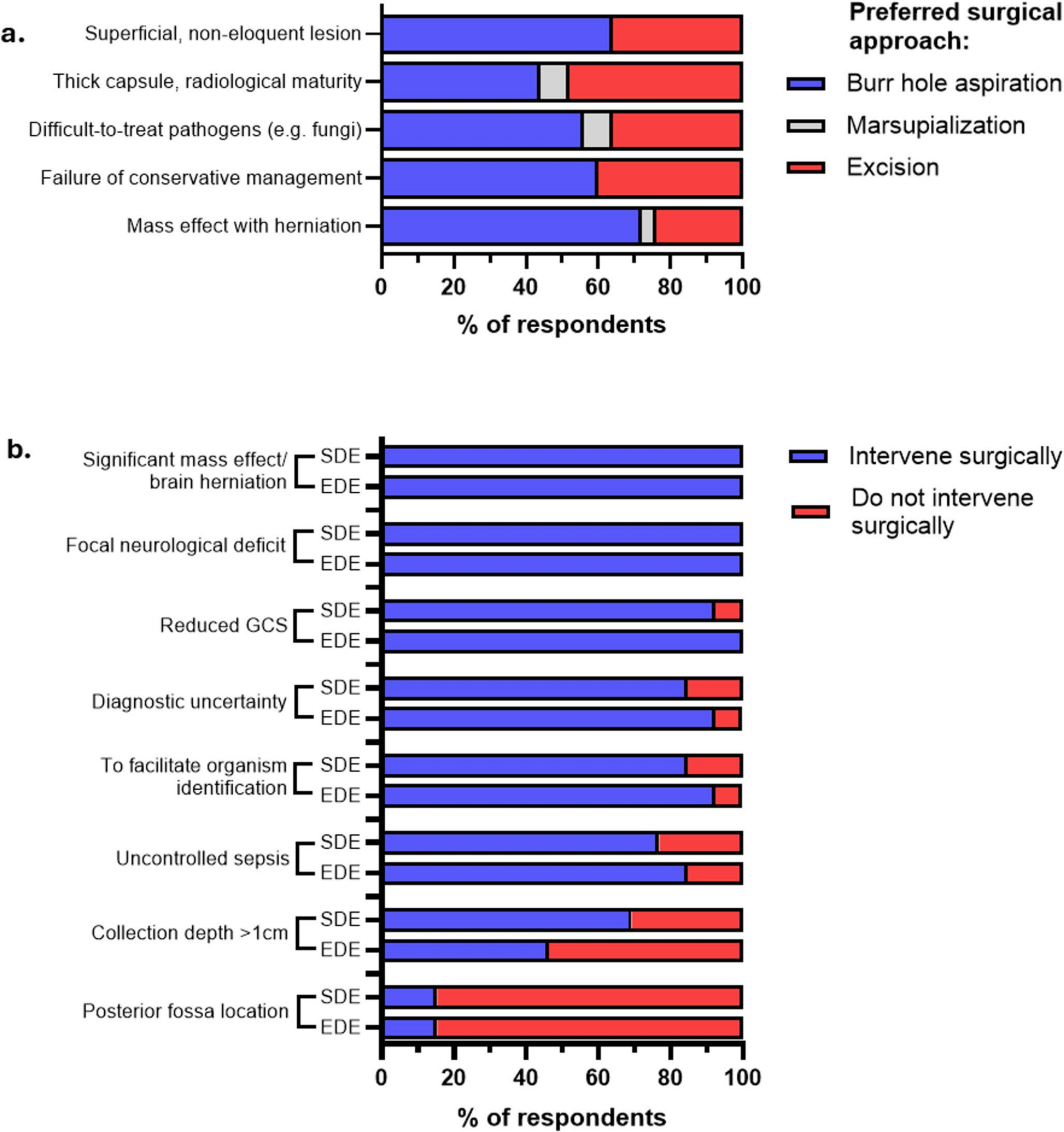
**a** Reported surgical approaches by underlying clinical scenarios. Stacked bars represent the proportion of respondents selecting each surgical approach for each scenario. Percentages correspond to the proportion of total responses **b** Respondent decisions regarding surgical intervention by underlying clinical scenarios in cases of subdural empyema (SDE) and epidural empyema (EDE). Stacked bars represent the proportion of respondents choosing to surgically intervene or to not surgically intervene. Percentages correspond to the proportion of total responses for each scenario

Most (22/25, 88%) responding NS entirely or mostly agreed that neurosurgical intervention is indicated for any BA *≥* 2.5 cm in diameter, irrespective of location, in a patient suitable for a general anaesthetic. If all options were technically feasible, most (21/25, 84%) NS favoured burr hole aspiration in BA, although this varied somewhat by clinical scenario (Fig. 2a). For patients with multiple abscesses, the majority (14/25, 56%) would operate on the largest lesion.

### SDE/EDE surgery

12/25 (48%) of NS would intervene surgically for SDE or EDE in all circumstances. Among those who would not routinely operate, there was broad consensus on most indications, but opinions were divided for collections with depth of >1 cm and only a minority would operate on posterior fossa empyema (Fig. 2b). The preferred surgical approach for SDE was craniotomy and washout (19/25, 76.0%). Craniectomy would be considered in some circumstances by 20/25 (80%), mainly if there was suspicion of osteomyelitis or if indicated for decompression (17/25, 68.0%). In the context of SDE and sinusitis, 11/25 (44.0%) reported that the ENT team always perform a sinus procedure alongside neurosurgery, although it was not clear in the data whether this was at the time of the neurosurgical operation, or shortly afterwards under a different anaesthetic.

### Adjunctive therapies

In the context of ruptured BA with ventriculitis, most (72/76, 94.7%) respondents would consider using intrathecal antibiotics. Experience with intracavitary antibiotics infiltrating directly into the abscess cavity was reported by 7/59 (11.9%) IS and 4/25 (16.0%) NS, amongst those who provided details individuals reported the use of intracavitary vancomycin, gentamicin and amphotericin/Ambisome.1/59 (1.7%) IS and 3/25 (12.0%) NS reported using steroids in all BA patients while 25/59 (42.4%) IS and 15/25 (60.0%) NS reported use in select patients, for example if there is significant oedema. 16/59 (27.1%) IS and 4/25 (16.0%) NS reported avoiding steroids, the remainder were ambivalent. Dexamethasone was most commonly reported as the steroid agent of choice. Amongst NS, a minority of 4/25 (16.0%) would use dural substitutes in managing brain abscesses.

### Imaging

**Fig. 3:**
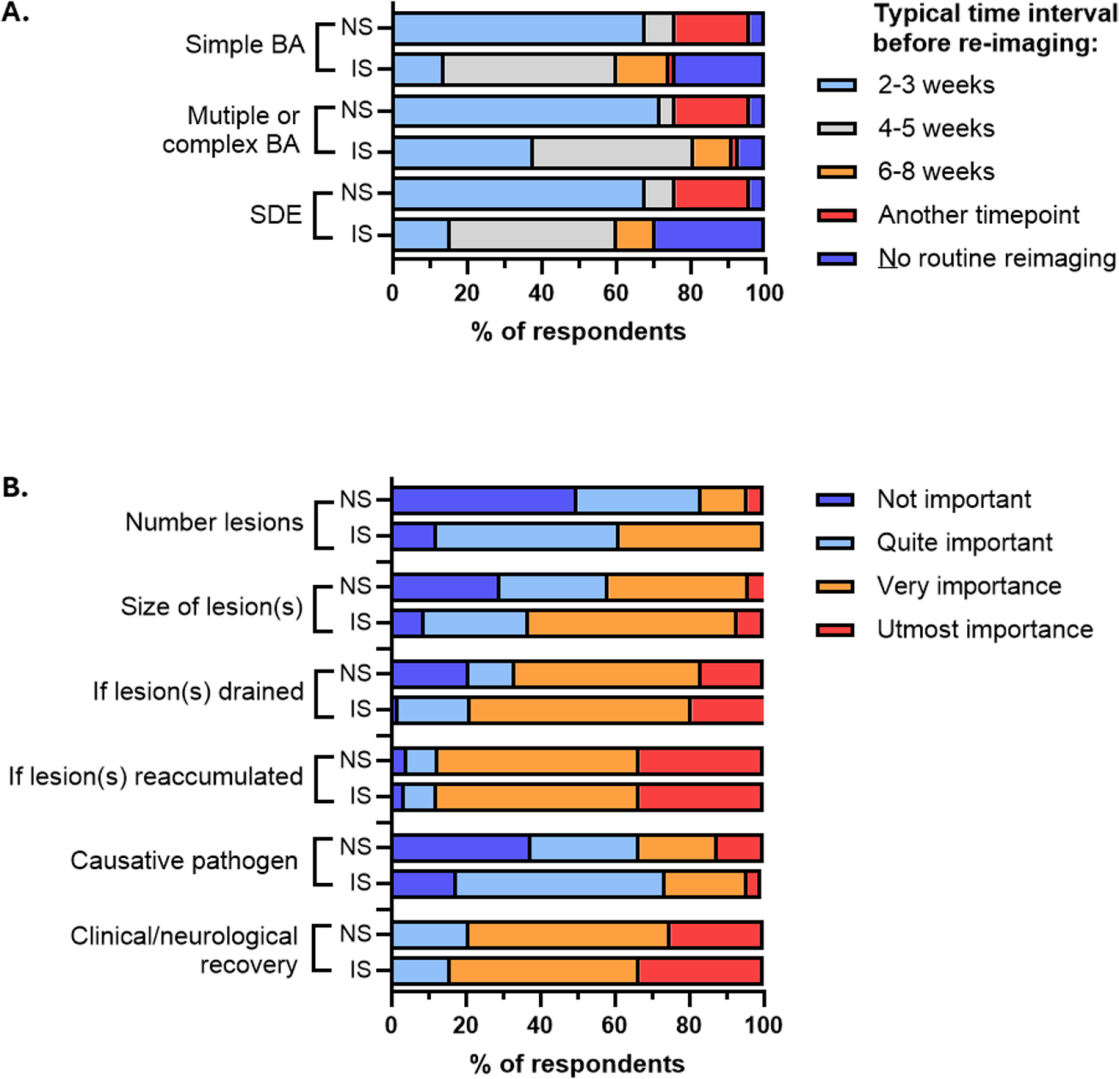
**a** Reported re-imaging timeframes by underlying clinical scenario by infection specialists (IS) and neurosurgeons (NS). Stacked bars represent the proportion of respondents reporting each re-imaging timeframe in in each scenario. Percentages correspond to the proportion of total responses **b** Importance of clinical considerations in determining re-imaging timeframe. Stacked bars represent the proportion of respondents rating each consideration when deciding on re-imaging timeframe. Percentages correspond to the proportion of total responses

The majority of respondents reported routinely reimaging patients with ICSI. There was a notable preference for repeat imaging, and in a shorter timeframe, for NS compared to IS (Fig. 3a). There was broad agreement on key factors for re-imaging, particularly clinical/neurological recovery, whether the lesion was drained and if it reaccumulated (Fig. 3b). Other considerations included input from other MDT members, lesion location, patient immunological status, antimicrobial resistance, or suspicion of atypical, mycobacterial, or fungal organisms. Cerebral angiography or venography in the context of SDE was only routinely requested by 7/56 (12.5%) IS, of which, 6/7 (85.7%) worked with paediatric patients, either exclusively or as well as adults. Almost all respondents, 24/25 (96.0%), reported routinely reimaging patients at the end of treatment.

## Discussion

The survey revealed that while there are areas of consistent practice in the UK management of ICSI, there is notable variability and clinical uncertainty. There is a strong consensus on empirical antibiotic choice, however, there is marked divergence in the duration of IV treatment. The preferred neurosurgical approach for BA is burr hole aspiration, and for SDE craniotomy and washout. However, there are multiple nuances in surgical practice. There is variation in the use of IT antibiotics, limited experience in the use of intracavitary antimicrobials and uncertainty around the use of corticosteroids. Respondents highlighted the role of MDT discussion in individualizing decision making around treatment and imaging. The diversity in practice in the management of ICSI highlights the need for the collection of granular primary data across centres, for example through the establishment of a prospective registry of case. This would allow for detailed exploration of the variables influencing clinical decision making, supporting the subsequent development of algorithmic management guidelines.

The consistent choice of empirical IV antibiotic was aligned with ESCMID guidelines: a third-generation cephalosporin, mostly alongside metronidazole (oral or IV). However, there was marked discrepancy in the duration of IV therapy. IS seeing a higher volume of BA cases more frequently reported short (1–3 week) typical durations of IV antibiotics than those seeing fewer cases. Early switch is contingent on sensitivity to a suitable oral antibiotic, with good absorption and CNS penetration, however, even if these circumstances evidence for shorter IV antibiotic courses in ICSI remains scant and UK clinical practice may have evolved independent of a specific evidence base. A recent UK study of paediatric ICSI patients identified that longer durations (*≥* 4 weeks) of IV antibiotics were not associated with improved outcomes and increased the risk of antibiotic-induced neutropenia [23]. The use of corticosteroids remains contentious, and there is limited experience in the use of intra-cavitary antibiotics. Respondents raised questions regarding the use of prophylactic anti-epileptics, recommended against in the ESCMID guidelines.

There are no clear neurosurgical guidelines for the management of BA/SDE which could explain current variability in practice and preference. Although general preference in BA was to perform burr hole aspiration, surgical decision making in BA and SDE was influenced by multiple factors including lesion size and location. In future work to develop guidelines support surgical decision making, detailed prospective outcome data including information about lesion size, eloquent/non eloquent location, supratentorial/infratentorial location, organism type and general features about the patient’s presenting age, Glasgow Coma Scale (GCS), frailty and systemic disease burden should be collected, alongside the surgical strategy used in each case. In SDE, the presence of osteomyelitis or the need for surgical decompression were likely to shift surgeon preference from craniotomy to craniectomy. Availability of ENT surgeons to operate alongside neurosurgeons during the primary neurosurgical procedure was variable and was raised as an important issue for some respondents. Only a small number of respondents indicated that they would operate on a posterior fossa empyema. This result needs further exploration in future studies. The response is likely to reflect the reality of highly nuanced decision making in this area which was not reflected in the survey question. Many empyemas in this region will be small and otogenic and an organism can therefore be identified following middle ear drainage. In other cases, multiple factors including patient age, co-morbidities, empyema depth and associated mass effect would need to be considered to facilitate the decision to operate or not.

There was discrepancy in the preferred approach to repeat brain imaging between IS and NS. ESCMID BA guidelines to do not include specific imaging timelines and this represents another important area for future consensus building. Despite of the association of ICSI with cerebral thrombosis, particularly in children [24, 25], few IS or NS reported routinely undertaking angiography. The role of dedicated angiography in managing these infections merits future study, as do approaches to anticoagulation in patients with ICSI and cerebral thrombosis who may need further neurosurgery.

Decision-making support varied across centres. A significant minority of respondents lack local treatment guidelines, and almost half do not discuss ICSI cases at an MDT meeting. This is challenging given the need to individualise decision making around IV antibiotic duration and repeat brain imaging timing was highlighted. Only one centre reported ENT involvement in MDTs, despite the association between sinusitis and ICSI and the need for ENT-led interventions in such cases [26].

The majority of UK neurosurgical centres participated in this survey, and respondents reported substantial relevant experience. A significant limitation is the lack of representation of Northern Ireland and limited representation of Scotland. Many centres were represented by a single respondent so results may not reflect diversity of practice within institutions. In addition, this survey could not capture the full scope of potential variables influencing clinical decision making. Whilst these limitations are not insignificant, we believe the results are as representative of UK clinical practice as is practically feasible in the absence of a mandatory national registry and identify key areas of consensus and discordance in current practice.

Areas for future work highlighted in the free text portion of the survey included the need for microbiological surveillance of causative organisms and the role of molecular diagnostic techniques such as PCR and metagenomics in microbiological diagnostics in ICSI. Several clinicians emphasised the value of gaining insights from their colleagues’ practice in this area given the relative rarity of, and high morbidity and mortality associated with, these conditions.

## Conclusions

This study identifies key areas of consensus (choice of antibiotic), variation (duration of IV antibiotic treatment, surgery for SDE, repeat brain imaging) and uncertainty (use of steroids) in the management of ICSI. There is pressing need for local guidelines and multidisciplinary support for decision making in many UK neurosurgical centres. There was clear interest amongst respondents for future work to develop a stronger evidence base and consensus-based guidelines to support decision making in the management of these infections.

## Supplementary Information

Below is the link to the electronic supplementary material.

Supplementary Material 1

## Data Availability

Anonymised raw data are available from the corresponding author on reasonable request.
