# Supplementary material for: Management of community-acquired brain abscess and intracranial empyema: a survey of UK neurosurgical centres
Source: Eur J Clin Microbiol Infect Dis. 2026 May 4;45(8):2521–9. doi: 10.1007/s10096-026-05522-4 (PMC13428759; doi:10.1007/s10096-026-05522-4)
Supplement: Supplementary file 1 — Supplementary Material 1 [file 10096_2026_5522_MOESM1_ESM.pdf]

Management of suppurative intracranial infection

Respondent

What is your medical specialty? (select all that apply)	☐ Infectious diseases (adult or paediatric)<br>☐ Microbiology<br>☐ Neurology<br>☐ Neurosurgery
How long have you been in this specialty?	◯ < 5 years<br>◯ 5-10 years<br>◯ 11-20 years<br>◯ >20 years
Do you work with adult patients, paediatric patients or both?	◯ Adult<br>◯ Paediatric<br>◯ Both
What region of the UK do you work in?	◯ Scotland<br>◯ Northern Ireland<br>◯ Wales<br>◯ North East England<br>◯ North West England<br>◯ Yorkshire and The Humber<br>◯ East Midlands<br>◯ West Midlands<br>◯ East of England<br>◯ London<br>◯ South East<br>◯ South West

What is your local neurosurgical centre?	◯ Aberdeen: Aberdeen Royal Infirmary<br>◯ Aberdeen: Royal Aberdeen Children's Hospital<br>◯ Belfast: Royal Victoria Hospital<br>◯ Birmingham: Birmingham's Children's Hospital<br>◯ Birmingham: Queen Elizabeth Hospital<br>◯ Brighton: Royal Sussex County Hospital<br>◯ Bristol: Bristol Royal Hospital for Children<br>◯ Bristol: Southmead Hospital<br>◯ Cambridge: Addenbrooke's Hospital<br>◯ Cardiff: University Hospital of Wales<br>◯ Coventry: University Hospital Coventry<br>◯ Dundee: Ninewells Hospital<br>◯ Edinburgh: Royal Hospital for Sick Children<br>◯ Edinburgh: Royal Infirmary<br>◯ Glasgow: Queen Elizabeth University Hospital (Institute of Neurological Sciences)<br>◯ Glasgow: Royal Hospital for Children<br>◯ Hull: Hull Royal Infirmary<br>◯ Lancashire: Royal Preston Hospital<br>◯ Leeds: Leeds General infirmary<br>◯ Liverpool: Alder Hey Children's NHS Foundation Trust<br>◯ Liverpool: The Walton Centre (Aintree University Hospital)<br>◯ London: Imperial College Healthcare (Charging Cross/St. Mary's)<br>◯ London: Great Ormond Street Hospital for Children<br>◯ London: King's College Hospital<br>◯ London: National Hospital for Neurology & Neurosurgery<br>◯ London: Royal London Hospital<br>◯ London: St George's Hospital<br>◯ Manchester: Royal Manchester Children's Hospital<br>◯ Middlesborough: James Cook University Hospital<br>◯ Newcastle: Royal Victoria Infirmary<br>◯ Nottingham: Queen's Medical Centre<br>◯ Oxford: John Radcliffe Hospital,<br>◯ Plymouth: Derriford Hospital<br>◯ Romford: Queen's Hospital<br>◯ Salford: Salford Royal Hospital<br>◯ Sheffield: Royal Hallamshire Hospital<br>◯ Sheffield: Sheffield Children's Hospital<br>◯ Southampton: Southampton General Hospital<br>◯ Stoke on Trent: Royal Stoke University Hospital
Approximately how many patients with brain abscesses do you routinely treat in your unit per year?	◯ 1-2<br>◯ 3-5<br>◯ More than 5
Approximately how many patients with subdural empyema do you routinely treat in your unit per year?	◯ 1-2<br>◯ 3-5<br>◯ More than 5

Local Approach

Do you have neurosurgery on site?	◯ Yes<br>◯ No
Do you have a local antibiotic guideline for the treatment of either brain abscess or subdural empyema?	◯ Both brain abscess and subdural empyema<br>◯ Brain abscess only<br>◯ Subdural empyema only<br>◯ No antibiotic guideline for either
Are brain abscess or subdural empyema cases discussed at a local MDT?	◯ All cases are discussed at an MDT<br>◯ Some cases are discussed at an MDT<br>◯ Cases are not discussed at an MDT
Who attends the MDT where these cases are discussed?	☐ Microbiology<br>☐ Infectious Diseases<br>☐ Neurology<br>☐ Neurosurgery<br>☐ Radiology/neuroradiology<br>☐ ENT<br>☐ Ophthalmology

Empiric Antibiotic Regimen

All questions relate to community-acquired brain abscesses with typical organisms (not TB, fungi or toxoplasmosis), in the absence of ventriculitis.

Please choose your typical initial empiric IV antibiotics for community-acquired brain abscess in immunocompetent patients from the list below (please choose all agents you would recommend/prescribe)

- ☐ Benzylpenicillin
- ☐ Cefotaxime
- ☐ Ceftriaxone
- ☐ Ceftazidime
- ☐ Meropenem
- ☐ Metronidazole
- ☐ Vancomycin
- ☐ Other

Which other empiric antibiotic would you recommend/prescribe?

____

Please describe the dosing regimen you would use for the antibiotics you have selected

____

If the patient had community-acquired subdural empyema rather than an abscess, would this alter your choice of initial empiric treatment?

- ◯ Yes
- ◯ No

Please describe how the empiric antibiotic(s) and dosing regimen you use for empiric treatment of community-acquired subdural empyema differ from that you use in community-acquired brain abscess

____

How long do you typically treat patients with the conditions below with IV antibiotics?

All questions relate to cases caused by typical bacterial organisms (not TB, fungi or toxoplasmosis), in the absence of ventriculitis with an uncomplicated recovery

	1 week	2 - 3 weeks	4 - 5 weeks	6 - 7 weeks	8 or more weeks
Conservatively managed brain abscess (not aspirated nor excised)	◯	◯	◯	◯	◯
Aspirated brain abscess	◯	◯	◯	◯	◯
Excised brain abscess	◯	◯	◯	◯	◯
Washed out subdural empyema	◯	◯	◯	◯	◯
Sinus-related subdural empyema in children managed without a neurosurgical procedure	◯	◯	◯	◯	◯

How much important are the following factors in your decision regarding the duration of IV antibiotics?

Again, all questions relate to cases caused by typical bacterial organisms (not TB, fungi or toxoplasmosis), in the absence of ventriculitis, with an uncomplicated recovery

	Not important	Quite important	Very important	Utmost importance
Number of lesions	◯	◯	◯	◯
Size of lesion(s)	◯	◯	◯	◯
If lesion(s) drained or not	◯	◯	◯	◯
If lesion has reaccumulated/recurred	◯	◯	◯	◯
Causative pathogen	◯	◯	◯	◯
Clinical or neurological recovery	◯	◯	◯	◯

What, if any, other factors influence your decision on the duration of IV antibiotics?

____

Do you ambulate suitable patients with community-acquired brain abscess or subdural empyema while on IV antibiotics?

◯ I ambulate suitable patients with either brain abscess or subdural empyema
◯ I ambulate suitable patients with brain abscess only
◯ I ambulate suitable patients with subdural empyema only
◯ I do not ambulate patients with either condition

Oral antibiotics

For patients with uncomplicated brain abscess or subdural empyema (no antibiotic allergies, toxicity issues, or renal/hepatic impairment)

Do you ever stepdown to oral antibiotics before completion of 6 weeks of IV antibiotics if:

A fully sensitive typical organism has been identified	◯ Yes<br>◯ No
NO organism has been identified	◯ Yes<br>◯ No
I sometimes stepdown to oral antibiotic regimen before completion of 6 weeks of IV antibiotics for patients with:	◯ Either brain abscess or subdural empyema<br>◯ Brain abscess only<br>◯ Subdural empyema only
What is the minimum duration of IV antibiotics you would consider using for a small (< 2.5cm), single, simple community-acquired brain abscess in an immunocompetent patient which has been aspirated or excised?	◯ 1 week<br>◯ 2 weeks<br>◯ 3 weeks<br>◯ 4 weeks<br>◯ 5 weeks
What is the minimum duration of IV antibiotics you would consider using for a simple community-acquired subdural empyema in an immunocompetent patient which has been washed out?	◯ 1 week<br>◯ 2 weeks<br>◯ 3 weeks<br>◯ 4 weeks<br>◯ 5 weeks

Oral antibiotic regimen

What duration of oral antibiotics would you use if you stepdown early?

- ◯ 1 week
- ◯ 2 weeks
- ◯ 4 weeks
- ◯ 6 weeks
- ◯ 8 weeks
- ◯ >8 weeks
- ◯ Dependent on the duration of IV therapy

How do you determine the planned duration of oral antibiotics?

____

Which oral antibiotic(s) and dosing regimen would you usually use for early stepdown treatment for:

Do you usually continue with oral antibiotic consolidation after completion of a "full course" of IV antibiotics for patients with brain abscess or subdural empyema?

- ◯ Yes in both brain abscess and subdural empyema
- ◯ Yes, in brain abscess only
- ◯ Yes, in subdural empyema only
- ◯ No, I do not use oral antibiotic consolidation

Which oral antibiotic(s) and dosing regimen do you usually use for consolidation treatment?

____

Brain abscess surgery

All questions relate to community-acquired brain abscesses with typical organisms (not TB, fungi or toxoplasmosis), in the absence of ventriculitis.

What is your preference in the neurosurgical management of brain abscesses, assuming all options are technically feasible?	◯ Burr hole aspiration<br>◯ Excision with resection of the abscess capsule<br>◯ Marsupialization
Neurosurgical intervention is indicated for any brain abscess (with or without capsule formation) that measures at least 2.5 cm in diameter, irrespective of location, in a patient suitable for a general anaesthetic.	◯ Entirely agree<br>◯ Mostly agree<br>◯ Mostly disagree<br>◯ Entirely disagree
In a patient with multiple brain abscesses would you normally operate on:	◯ All lesions > 2.5cm in size<br>◯ The largest lesion<br>◯ Other
Please elaborate on which abscesses you would normally operate on in this context	__________________________________
In subdural empyema or brain abscess in the context of sinusitis, do ENT do a sinus drainage procedure alongside neurosurgical intervention?	☐ Yes always<br>☐ Sometimes<br>☐ No
In what context would ENT perform sinus surgery alongside neurosurgery?	__________________________________

Which neurosurgical approach is preferred in the following contexts? (Assuming all are technically feasible)

	Burr hole aspiration	Neurosurgical excision with resection of the abscess capsule	Marsupialization
Superficial lesions NOT located in the eloquent areas of the brain?	○	○	○
When the abscess produces mass effect leading to brain herniation	○	○	○
When the abscess capsule appears thick, and the abscess appears radiologically "mature"	○	○	○
Isolation of a difficult to treat pathogen, for example fungi	○	○	○
Failure of conservative management (treatment with antibiotics alone)	○	○	○

Subdural empyema surgery

Under what circumstances would you routinely intervene surgically for subdural empyema? (select all relevant options)

- ☐ In all cases
- ☐ Reduced GCS
- ☐ Significant mass effect and/or brain herniation
- ☐ Depth>1cm
- ☐ Posterior fossa location
- ☐ Focal neurological deficit
- ☐ Uncontrolled sepsis
- ☐ Diagnostic uncertainty
- ☐ To facilitate organism identification

What is your preferred surgical approach to subdural empyema, assuming all are technically feasible?

- ◯ Burr hole evacuation
- ◯ Craniotomy and washout
- ◯ Craniectomy and washout

What is your usual practice with regards to craniectomy when performing a craniotomy for subdural empyema?

- ◯ I would routinely perform a craniectomy regardless of whether there is suspicion of osteomyelitis
- ◯ I would routinely perform a craniectomy only if there is suspicion of osteomyelitis or if indicated for decompression
- ◯ I would only perform a craniectomy if indicated for decompression

Under what circumstances would you routinely intervene surgically for extradural empyema? (select all relevant options)

- ☐ In all cases
- ☐ Reduced GCS
- ☐ Significant mass effect and/or brain herniation
- ☐ Depth>1cm
- ☐ Posterior fossa location
- ☐ Focal neurological deficit
- ☐ Uncontrolled sepsis
- ☐ Diagnostic uncertainty
- ☐ To facilitate organism identification

Adjunctive therapies

Do you have any experience with the use of intracavitary antibiotics in the management of brain abscess?

◯ Yes
◯ No

Please describe the approach used (including agent and dose if possible) in the use of intracavitary antibiotics?

____

What is your practice regarding dural substitutes for dural repair in the context of brain abscesses:

◯ Would use
◯ Would not use

In the context of an abscess with intraventricular rupture and ventriculitis, please indicate your practice with regards to intrathecal antibiotic treatment

◯ I would never administer/recommend intrathecal antibiotics
◯ I would sometimes administer/recommend intrathecal antibiotics
◯ I would always administer/recommend intrathecal antibiotics

Regarding steroids in brain abscess, which statement best fits your practice:

◯ I avoid using steroids in all patients with a brain abscess
◯ I am ambivalent about the use of steroids in brain abscess
◯ I use steroids in select patients with brain abscess (for example, if there is significant oedema around the lesion)
◯ I use steroids in all patients with brain abscess

If/when you prescribe steroids for brain abscess, please describe which steroid and dosing regimen you would use

____

Repeat Imaging During Treatment:
Would you routinely re-image during treatment patients with the following (assuming a straightforward recovery with no complicating factors), and if so when:

	I wouldn't routinely reimage during treatment	I would reimage at 2 - < 4 weeks	I would reimage at 4 - < 6 weeks	I would reimage at 6 - 8 weeks	I would reimage at another timepoint
Single simple brain abscess	○	○	○	○	○
Multiple or complex brain abscesses	○	○	○	○	○
Subdural empyema	○	○	○	○	○

When would you reimage a patient with a single simple brain abscess? ______________________________________

When would you reimage a patient with multiple or complex brain abscesses? ______________________________________

When would you reimage a patient with subdural empyema? ______________________________________

In patients with subdural empyema would you routinely request cerebral angiography/venography?
○ Yes
○ No

When deciding if and when to reimage, how important are the following?

	Not important	Quite important	Very important	Utmost importance
Number of lesions	◯	◯	◯	◯
Size of lesion(s)	◯	◯	◯	◯
If lesion(s) drained or not	◯	◯	◯	◯
If lesion(s) reaccumulated/recurred	◯	◯	◯	◯
Causative pathogen	◯	◯	◯	◯
Clinical or neurological recovery	◯	◯	◯	◯

What, if any, other factors influence the imaging schedule you use?

____

Do you routinely reimage patients at the end of treatment?

◯ Yes in both brain abscess and subdural empyema
◯ Yes, in brain abscess only
◯ Yes, in subdural empyema only
◯ I do not routinely reimage at the end of treatment

Thank you for your time in completing this survey

Are there any questions regarding the management of brain abscess or subdural empyema that we have not included that you would like to see addressed? __________________________________
